# Occurrence, Bioaccumulation, and Trophic Transfer of Short-Chain Chlorinated Paraffins (SCCPs) in a Marine Food Web from Laizhou Bay, Bohai Sea (Eastern China)

**DOI:** 10.3390/toxics12120877

**Published:** 2024-11-30

**Authors:** Min Song, Dianfeng Han, Shunxin Hu, Qingkui Cui, Huanjun Li, Fan Li, Jianbai Zhang, Yongchun Liu, Mei Zhao, Cunxin Zhang, Yingjiang Xu

**Affiliations:** 1 College of Food Sciences & Technology, Shanghai Ocean University, Shanghai 200120, China; 2 Shandong Provincial Key Laboratory of Restoration for Marine Ecology, Shandong Marine Resource and Environment Research Institute, Yantai 264006, China; 3 Yantai Marine Economic Research Institute, Yantai 264003, China

**Keywords:** emerging pollutants, bioaccumulation, trophic transfer, Bohai Sea

## Abstract

Short-chain chlorinated paraffins (SCCPs) are a persistent organic pollutant, and limited information is available on their bioaccumulation and trophic transfer, which would be affected by carbon chain length, chlorine content, and hydrophobicity. In this study, relevant data on SCCPs in water, sediments, and organisms collected from Laizhou Bay were analyzed to investigate the specific distribution of SCCPs and their bioaccumulation and trophic transfer. In water and sediments, the average SCCP concentrations (ΣSCCPs) were 362.23 ± 81.03 ng/L and 609.68 ± 90.28 ng/g d.w., respectively. In 28 species of organisms, the ΣSCCPs varied from 70.05 to 47,244.13 ng/g l.w. (average = 648 ± 7360) and the predominant homologs were C_13_ (average = 34.91%) and Cl_5–7_ (average = 93.13%), differing from those in water (average = C_11_ 32.75% and average = Cl_5–7_ 88%) and sediments (average = C_13_ 31.60% and average = Cl_6–8_ 87.16%). The logarithm bioaccumulation factors (BAFs) of ΣSCCPs were 1.18–2.74 and were positively correlated with the log K_ow_. A significant negative linear relationship was observed between biota-sediment accumulation factors (BSAFs) and log K_ow_. It is suggested that the hydrophobicity may affect the bioaccumulation of SCCPs. SCCPs demonstrated a trophic magnification factor (TMF) ranging from 2.19 to 3.00 (average = 2.51) and exhibited a significant linear correlation with carbon chain length (*p* < 0.05) and log K_ow_ values (*p* < 0.05), suggesting that SCCPs have biomagnification potential in Laizhou Bay that is affected by hydrophobicity and carbon chain length.

## 1. Introduction

Short-chain chlorinated paraffins (SCCPs) have been widely used in industrial products such as polyvinyl chloride, coatings, and rubber. They are released into the environment during their production, use, and transport, as well as through leakage [1,2]. SCCPs were included in the Stockholm Convention on Persistent Organic Pollutants in 2017 [3]. Similar to traditional persistent organic pollutants (POPs), SCCPs exhibit hydrophobicity and long-range transport characteristics, allowing them to become widely distributed in various environmental matrixes [4,5]. The marine environment, a primary SCCP “sink”, has been significantly impacted by their accumulation. For example, the concentration range of SCCPs in seawater along the coast of the Bohai Sea in China was 572.6–1978 ng/L [6], and sediments in the South China Sea had a range of 133–716 ng/L d.w. [7]. Furthermore, the influence of climate [8,9] and ocean currents [10] have enabled researchers to discover SCCPs in polar organisms [11,12], indicating that SCCP pollution has become a problem of global significance.

SCCPs possess the capacity to bioaccumulate and become toxic. According to previous research, prolonged SCCP exposure may impact metabolism [13] and the endocrine system [14,15] and can induce neurotoxicity [16]. The unique chemical structure of SCCPs enables them to form lipid complexes in organisms and gradually accumulate within the body, which has been observed in aquatic environments in China [17,18,19]. Previous studies have indicated that fresh seafood is the primary dietary preference among coastal city populations and exhibits higher levels of SCCPs compared with other food categories [20,21]. SCCP accumulation in a species may pose potential risks to maternal health, predators, and humans. It is imperative to investigate the distribution of SCCPs in seafood products and analyze their potential hazards.

Previous studies have demonstrated the bioaccumulation and trophic magnification of SCCPs in aquatic food webs [17]. Our understanding of the mechanisms underlying the transfer and behavior of SCCPs in different media remains limited. Studies have shown that organisms can absorb, metabolize, and transform hydrophobic compounds found in sediments and seawater [22]. However, whether their unique feeding strategies, different living environments, and ecological characteristics regulate the in vivo distribution and homologous characteristics of SCCPs remains to be studied. In addition, although SCCPs can be transferred from prey to predators [23], reports on the mechanism of trophic transfer in organisms are inconsistent.

The trophic magnification factor (TMF) values in Pearl River [23] and Liaodong Bay [24] were 1.32–2.67 and 2.57, respectively, indicating that SCCPs could be amplified in these food webs; however, this contrasts with the researchers’ observations in some mollusks [25] and freshwater food webs that the average TMF values were 0.24 and 0.17, respectively [19]. The bioavailability, offspring genetics, environmental temperature, exposure level, and food web structure may affect SCCP accumulation in organisms. Dietary intake (EDI) is the primary route through which humans are exposed to SCCPs, and the presence of SCCPs has been detected in aquatic products [26]. Therefore, the above complex results indicate the necessity to further study the trophic transport behavior of SCCPs in different food webs and explore the main enrichment pathways and influencing factors of SCCPs in organisms.

Laizhou Bay (LZB), located in the south Bohai Sea in China, has become significantly contaminated in recent years because of the detrimental effects of industrial effluents, domestic sewage, and land-based river pollution resulting from rapid industrialization and urbanization [22]. Moreover, LZB is a semi-enclosed bay with poor water dynamics and has been affected by the pronounced accumulation of pollutants. Researchers have identified high levels of POPs, including polychlorinated diphenyl ethers [27], dioxins [28], and organophosphate esters [29], and analyzed their potential behavior of trophic transfer in the LZB food web. However, the analysis of SCCPs needs to be expanded. In this study, the surface seawater, sediments, and organisms of LZB were collected to analyze the concentration and homolog distribution of SCCPs. Based on the LZB food web model, the trophic transfer potential of SCCPs along different food chains was analyzed, and the influencing factors were discussed. A risk assessment of the SCCPs in LZB was conducted to improve related research studies.

## 2. Materials and Methods

### 2.1. Sample Collection

**Figure 1:**
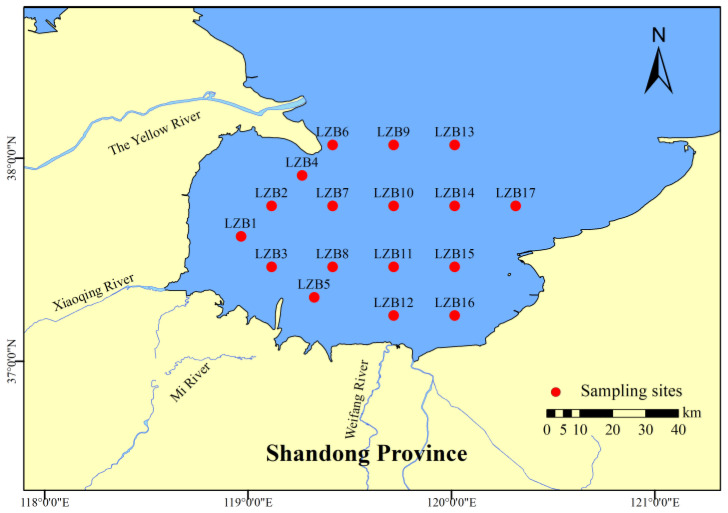
Sampling stations for surface water, sediments, and organisms in Laizhou Bay.

In October 2022, seawater, sediments, and 28 species of organisms were collected in LZB (Figure 1). Water samples were collected 50 cm below the surface with a water collector (Organic Glass Water Sampler, HYDRO-BIOS Company, DE) from each point. Sediments (10 cm) and benthic fauna were collected using a 0.1 m^2^ mud collector. In addition, larvae and juveniles were sampled using large plankton nets and swimmers were sampled using wing-bottom trawls and surface horizontal or vertical trawls; this was carried out in 10 min intervals at a tow rate of 3.7 km/h. All species ranged from 3 to 14 individuals in every station. The organisms were rinsed with Milli-Q water and body length and weight were recorded before they were stored at −80 °C until laboratory analysis (Table S1). In addition, combined with the research of Cao [30] and Wang et al. [31], these species were grouped based on their living environment and feeding habits (Table 1), and the scientific names and comment names of species are shown in Text S1.

### 2.2. Sample Pretreatments

The extraction and pretreatment methods of SCCPs from water, sediments, and organisms referred to the methods of previous studies with minor modifications [32]. Briefly, seawater was filtered using a glass fiber filter membrane (pore size 0.45 μm), and 500 mL of filtered seawater was added to 20 mL of 0.1% ammonia methanol (*v*/*v*) solution (pH 4.0) and extracted using a solid-phase extraction instrument (GX-274 ASPEC, Gilson Company, Middleton, WI, USA) with an HLB filter. This was subsequently eluted using 6 mL of dichloromethane/hexane (*v*/*v* = 1:1) and concentrated to 1 mL using a nitrogen gas flow at 40 °C (nitrogen blowing instrument: N-BTM112, Orangomation Associates Inc., MA, USA) for further purification. The freeze-dried sediments (dry weight = 2 g) and organisms (wet weight = 2 g) were extracted using 10 mL dichloromethane/hexane (*v*/*v* = 1:1), sonicated for 30 min, and centrifuged at 8000 rpm; this step was repeated twice. The resultant solution was concentrated from 12 mL to 1 mL using a nitrogen gas flow at 40 °C. Based on previous studies [33,34,35], these concentrated solutions were purified using a composite silica gel column containing 3.5 g anhydrous Na_2_SO_4_, 5 g acidic silica gel, 2 g silica gel, and 3 g Florisil. The eluate was collected, dried, and concentrated using a nitrogen gas flow at 40 °C. The volume was fixed with 1 mL acetonitrile, vortexed for 30 s, sonicated for 5 min, and then the solution was filtered through a 0.22 μm polyvinylidene fluoride (PVDF) filter membrane. In addition, the biological lipid content was determined using Soxhlet extraction.

### 2.3. Instrumental Analysis

SCCPs were analyzed using ultra-high performance liquid chromatography coupled with Q-Exactive Focus mass spectrometry (UPLC-Q-Exactive Focus-MS, Thermo Corporation, Waltham, MA, USA) according to the methods described by Cui [32] and Huang et al. [34]. The conditions were as follows: spray voltage of 2.5 kv, capillary temperature of 320 °C, injection volume of 3 μL, tetramethyl ammonium chloride (TMAC) concentration of 0.05 mM, flow rate of 0.3 mL/min. Ionization was performed using a negative Heated Electrospray Ionization (HESI) source with ultrapure water and acetonitrile as mobile phases. The acquisition parameters for SCCPs are shown in Table S2.

### 2.4. Quality Assurance and Quality Control (QA/QC)

The experimental apparatus was made of glass to reduce accidental contamination. The concentration gradient for the calibration curve was set to 10, 50, 100, 200, and 500 μg/L for analyzing the target compound, and the R values were greater than 0.998. The whole blank program for SCCPs in seawater, sediments, and organisms was below or close to the detection limit, which was 10.0 ng/L, 5.0 ng/g d.w., and 5.0 ng/g w.w., respectively. The recovery rates for SCCPs in seawater, sediments, and organisms were 21.25–30.06%, 74.91–86.65%, and 57.11–73.11%, respectively. The detection rates for all samples were 100%. SCCP standards (51.5%, 55.5%, and 63% chlorine content) were obtained from Dr. Ehrestorfer GmbH (Augsburg, Germany). The detailed information is shown in Text S2.

### 2.5. Stable Isotope Analysis and Calculations

#### 2.5.1. Trophic Level (TL)

The trophic level of organisms was determined based on stable isotopes [35]. A detailed description is given in Text S3. The trophic level of baseline organisms (TL_baseline_) was 2.0 and the trophic enrichment (ΔN) was 3.8‰ [36,37]. The δ^15^N_consumer_ and δ^15^N_baseline_ were the δ^15^N value of the consumer and baseline indicator. The calculation formula was as follows (Equation (1)).

(1) $$\mathrm{TL}={\mathrm{TL}}_{\mathrm{baseline}}+\left[\left(\delta^{15}N_{\mathrm{consumer}}-\delta^{15}N_{\mathrm{baseline}}\right)/\Delta N\right]$$

#### 2.5.2. Bioaccumulation and Trophic Transfer

The bioaccumulation factor (BAF) is the ratio of the SCCP concentration (∑SCCP) in an organism (*C*_biota_, ng/g w.w.) to the average concentration in the aquatic environment (*C*_water_, μg/L) and was calculated using Equation (2), which was used in a previous study [38].

(2) $$\mathrm{BAF}=C_{\mathrm{biota}}\times 1000/C_{\mathrm{water}}$$

The determination of the biota–sediment accumulation factors (BSAFs) was calculated using Equation (3).

(3) $$\mathrm{BSAF}=C_{\mathrm{infa}}/\left(C_{\mathrm{sediment}}\right./\left.\mathrm{TOC}\right)\;$$

where $C_{\mathrm{infa}}$ is the ∑SCCP (ng/g l.w.) in infauna organisms and $C_{\mathrm{sediment}}$ is the ∑SCCP in sediments (ng/g d.w.). In this study, the average total organic carbon (TOC) value was 1.22%.

The TMF value was calculated based on the relationship between the lipid-normalized ∑SCCPs and TLs in the marine food web using Equation (4).

(4) $$\log C_{\mathrm{biota}}=a+b\times \mathrm{TL}$$

where slope b was used to calculate the TMF value via Equation (5).

(5) $$\mathrm{TMF}=10^{b}$$

### 2.6. Dietary Risk

EDI is an important route of human exposure to SCCPs, among which, seafood is a primary contributor. The EDI of SCCPs for local residents was estimated using Equation (6).

(6) $$\mathrm{EDI}=\frac{C\times \mathrm{IR}}{\mathrm{BW}}$$

where C is the ∑SCCP in seafood (ng/g w.w.), IR is the average daily intake of seafood (g/day), and BW is the average body weight (kg) of local residents.

These data regarding aquatic product consumption were based on the daily consumption of aquatic food in different populations in two northern regions from *The Fifth Chinese Total Diet Study (CTDS)*, and the body weight of people of all ages was based on the average representative value of the Chinese population [39].

Hazard quotients (HQs) were applied to assess the health risks of SCCPs using Equation (7).

(7) $$\mathrm{HQ}=\frac{\mathrm{EDI}}{\mathrm{TDI}}$$

The tolerable daily intake (TDI) for SCCPs was 30 μg·kg^−1^·d^−1^ [40]. When HQ > 1, the current pollutant concentration was potentially risky.

### 2.7. Statistical Analysis

Prior to running ANOVA, the normality and homogeneity of variance in these data were tested using the Kolmogorov–Smirnov and Levene’s tests, respectively. These data met both assumptions and were analyzed using one-way ANOVA to examine the differences in the ∑SCCPs in environmental media or organisms from different areas. The ∑SCCPs in organisms were expressed as items per wet body mass (item/g w.w.), lipid weight (item/g l.w.), and dry weight (item/g d.w.). All data were expressed as the mean ± standard deviation (S.D.).

The significance level of difference or correlation was set at *p* < 0.05 in this study. Data analyses were conducted on SPSS 26.0 (IBM Corporation, Armonk, NY, USA), Excel 2021, and Origin 2021.

## 3. Results and Discussion

### 3.1. Homologs Profiles of SCCPs in Laizhou Bay

Based on previous studies, Chinese chlorinated paraffins (CPs) primarily consist of a mixture of CP-42, CP-52, and CP-70, characterized by chlorine contents of 42%, 52%, and 70%, respectively, with CP-52 comprising 80–90% of total CP production [1,41]. These products are known to release SCCPs during their production, transportation, and utilization. The proportions of SCCPs in these products are 3.1%, 40.2%, and 1.7%, respectively. Within CP-52, the congeners of C_10–13_ constitute 12.1–19.3%, 17.1–25.1%, 25.1–26.9%, and 21–41.33%. In CP-42 and CP-70, the proportions of C_10_ are 85.6% and 40.3%.

**Figure 2:**
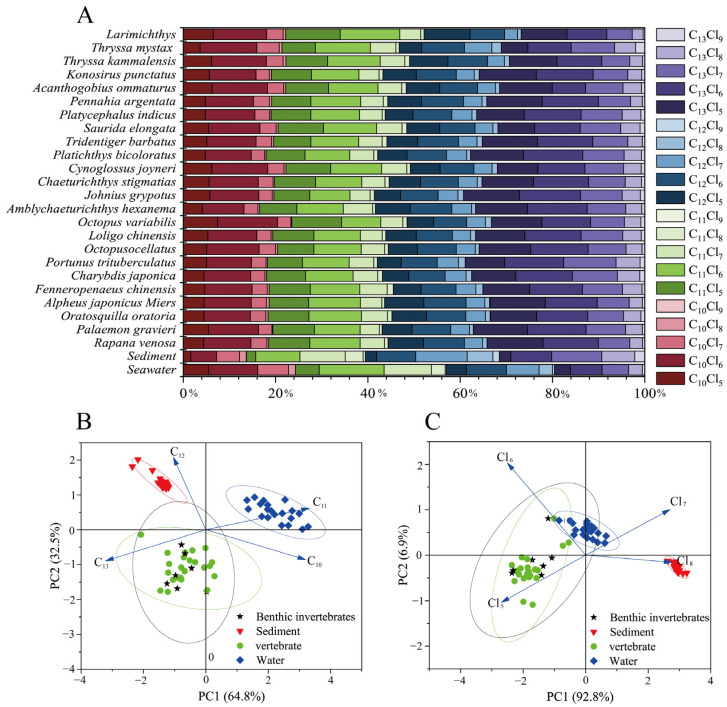
Abundance profile of SCCPs in seawater, sediments, and organisms (**A**); PCA of the number of carbon atoms (**B**) and chlorine atoms in SCCPs in seawater, sediments, and organisms (**C**).

In the present study, the observed carbon chain lengths in sediments followed the order C_13_ (32.75%) > C_12_ (29.06%) > C_11_ (25.69%) > C_10_ (12.5%), while the chlorine content was predominated by Cl_6–8_ (87.12%), consistent with the characteristics of CP-52. In contrast, in seawater, the carbon chain length C_11_ was the most abundant at 32.75%, followed by C_10_ at 24.00%, with Cl_5-7_ accounting for the highest chlorine content at 88.00% (Figure 2). This distribution may be attributable to the influence of hydrophobicity and molecular weight, as SCCPs with lower carbon chain lengths and chlorination levels exhibit higher solubility, while longer carbon chain homologs tend to accumulate in sediments. Furthermore, in seawater, the concentrations of C_12_ (23.45%) and C_13_ (19.80%) were higher than those in CP-42 and CP-70. This analysis indicates that the SCCPs present in the marine environment of LZB likely originated from CP-42, CP-52, and CP-70, with CP-52 being the predominant contributor.

In organisms, the distribution of carbon chain lengths was C_13_ (34.91%) > C_11_ (25.39%) > C_12_ (20.01%) > C_10_ (19.96%), consistent with findings from aquatic organisms in southern China [18]. This distribution may be influenced by the hydrophobicity and metabolic resistance of compounds, which are discussed in Section 3.3 and Section 3.4. Furthermore, the chlorine content was observed to follow the order: Cl_6_ (41.33%) > Cl_5_ (30.03%) > Cl_7_ (21.77%) > Cl_8_ (6.34%) > Cl_9_ (0.79%). This distribution can be attributed to the complex physicochemical reactions that occur when SCCPs enter an organism; the higher the Fukui index level of a Cl atom, the more vulnerable it is to attack in oxidation processes, and minimal chlorination of the SCCP can be achieved [42]. Additionally, the greater environmental abundance of Cl_6_ congeners compared with Cl_5_ congeners may account for the higher prevalence of Cl_6_ in organisms. Furthermore, the principal component analysis (PCA) revealed significant variations in the abundance of homologs among organisms, sediments, and seawater, particularly concerning carbon chain lengths, while no differences in species were observed (Figure 2B,C). This finding suggests that the distribution of SCCPs within the environmental matrix is likely influenced more by carbon chain length than chlorine content.

### 3.2. SCCP Concentrations in Organisms

**Figure 3:**
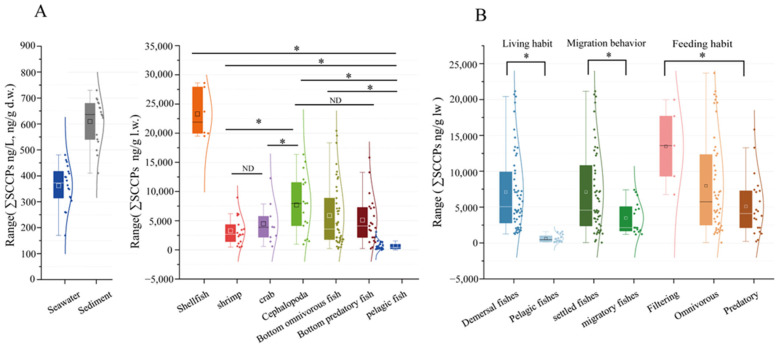
Comparison of SCCPs in samples obtained from LZB (**A**) and in different biological and ecological features (**B**). Note that “ND” indicates no difference between the variables and “*” indicates a significant difference, as does *p* < 0.05.

SCCPs were detected in all groups of organisms (Table 1). The concentrations ranged from 70.05 ng/g l.w. to 47,244.13 ng/g l.w., with an average of 6052.31 ± 7526.41 ng/g l.w. The ∑SCCPs in shellfish were significantly higher than in other organisms (*p* < 0.05, Figure 3A). In filter feeders (average = 11431.14 ± 1070.43 ng/g l.w.), the concentrations were significantly higher than in predators (*p* < 0.05, Figure 3B). These findings are similar to findings from the Pearl River Estuary [23]. They might be related to the living environment and feeding habits. In this study, shellfish fed mostly on organic detritus and plankton. In comparison, predation was not always present in the environment, which might have reduced their SCCP intake compared with filter-feeding organisms [43]. In addition, benthic organisms were more prone to accessing sediments and absorbing certain SCCPs through sediment ingestion during foraging activities. This may have caused the ∑SCCPs in pelagic fish (average = 664.34 ± 520.31 ng/g l.w.) to be significantly lower than in demersal fish (average = 6509.73 ± 5610.76 ng/g l.w.; *p* < 0.05, Figure 3B). These results suggested that the enrichment of SCCPs was affected by the feeding habits, living environment, and migratory behavior.

In addition, the distribution of SCCPs in species reflects differences in the source of SCCPs in marine environments. The ∑SCCPs in marine organisms were significantly higher in industrially developed areas compared with the surrounding remote areas. For example, the ∑SCCPs in Indo-Pacific humpback dolphins (2800 ng/g l.w.) residing near the estuary of the downstream Pearl River Delta exceeded that found in finless porpoises (1800 ng/g l.w.) inhabiting waters in southern Hong Kong [44]. Moreover, this study found that the ∑SCCPs in organisms in LZB were lower than in the Yellow River Estuary, which was tested previously [34] and is consistent with the findings of Huang et al. [17]. Additionally, as shown in Figure 3B and Table 1, the ∑SCCPs were higher in demersal sedentary fish compared with those in migratory spawning fish. This difference could be attributable to the fact that migratory fish inhabit a broader range of environments. Furthermore, during migration, seawater environments exhibit greater variability, indicating a higher impact of SCCPs in bay areas compared with open water. Therefore, SCCP levels could decrease with increasing distance from the coastline. This suggested that riverine inputs were the primary source of SCCPs in the marine environments. Therefore, the differential distribution of SCCPs in various environmental matrices could be used for source identification and emission monitoring in industrial settings.

### 3.3. Bioaccumulation and Biota-Sediment Accumulation of SCCPs

BAFs and BSAFs were used to indicate the bioaccumulation potential of organisms for SCCPs. In this study, as shown in Table 1, the log BAF values of ∑SCCPs ranged from 1.18 to 2.74 and were higher in benthic invertebrates (2.22) than in vertebrates (2.08), suggesting that benthic invertebrates have higher bioaccumulation potential than vertebrates. The values for SCCP homolog log K_ow_ refer to Sun et al. [19]. Among the benthic organisms, crabs exhibited the highest BAF (2.50), which was similar to the results of the South China Pearl River [17] and East China Sea [45], potentially attributable to their predominant habitat preference of residing beneath sand and rocks. The BAF was also influenced by the size of the species, their activity level, and energy [46]. Pelagic fishes (2.34) had a higher BAF than benthic organisms (2.08), probably because of differences in the living environment and the higher lipid contents. In this study, the ∑SCCPs in organisms, on a wet weight basis, had a significant positive correlation with the lipid content (*p* < 0.05, Figure S1), indicating that lipophilicity may be a key factor in the bioaccumulation of SCCPs.

In general, the log BAF of SCCPs had a significant positive correlation with log K_ow_ [17,18,19,24,46], similar to this study (*p* < 0.05, Figure S4A). In the pelagic fishes *Thryssa kammalensis* and *Thryssa mystax*, significant linear relationships were observed (*p* < 0.05, Figure S4B). This phenomenon may be attributable to the fact that most species in this study inhabited demersal living habits [30] that were more susceptible to sediment exposure and the partial uptake of SCCPs from these sediments. In addition, the lipid content in these fish was higher than in the other organisms in this study. According to a previous report, small hydrophobic congeners primarily undergo passive diffusion from the aqueous phase into the lipid membrane [47]. During this process, the binding affinity between POPs and lipids increased gradually in correlation with an elevated log K_ow_ and a higher lipid content [47]; therefore, hydrophobicity may be a key factor in the BAF for SCCPs. Moreover, the differences in living habits among species and the lipoatrophy of target compounds could also have an influence. Additionally, in this study, a significant positive relationship with carbon chain length was observed (*p* < 0.05, Figure S2). In contrast, a negative relationship with chlorine content (*p* < 0.05, Figure S3) was observed with regard to BSAFs (*p* < 0.05, Figure S6), suggesting that chlorine content may be the main factor limiting the bioaccumulation of SCCPs.

In the sediment–organism pathway, the BSAF had a significant negative correlation with log K_ow_ (*p* < 0.01, Table S3), which was contrary to log BAF. This may have been because the congeners with a high log K_ow_ had a higher affinity for organic matter in sediments than lipids, making them more likely to accumulate in sediments [10]. Furthermore, organic compounds, which can coexist with SCCPs, may have been present in these sediments and could combine with SCCPs to affect the potential for bioaccumulation by reducing the free soluble ∑SCCP concentration and inhibiting the biological availability of extremely hydrophobic congeners [48]. These results indicated that hydrophobic congeners could largely determine the bioaccumulation and biota–sediment accumulation of SCCPs, and the compounds that exist in the environment matrix may interfere with this process.

### 3.4. Trophic Transfer of SCCPs in the Food Web

**Figure 4:**
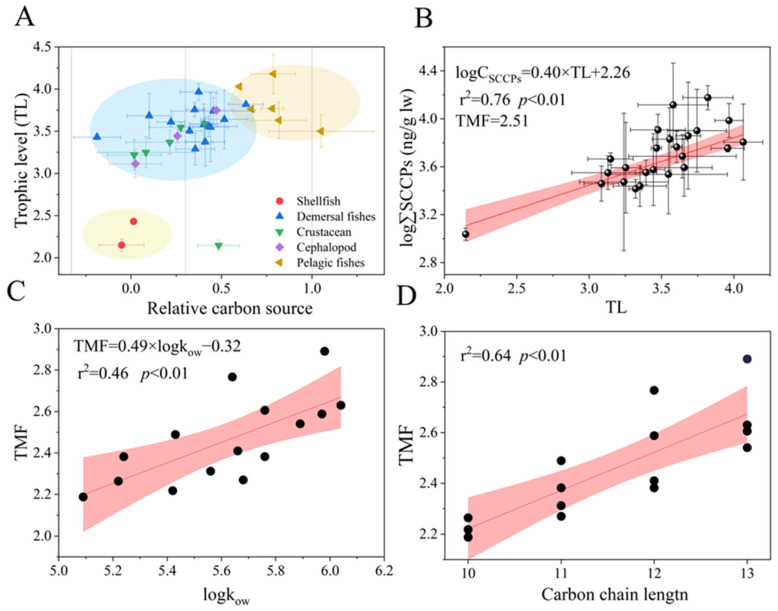
Relative carbon source and trophic levels characterized the food web relationship (**A**). Relationship between TL and log-transformed concentrations of the SCCPs (ng/g l.w.) (**B**) and the relationships of TMFs of the SCCP congener group with logKow (**C**) and the carbon chain length (**D**). Note: The red section indicates the 95% confidence interval.

The δ^13^C and δ^15^N values of organisms are shown in Table S4. A trend was found, following the order pelagic fish (13.76‰) > demersal fish (13.48‰) > cephalopods (12.75‰) > benthic crustaceans (11.81‰) > shellfish (8.07‰) > zooplankton (7.44‰) (Figure S7). The values for relative carbon sources varied from 0.19 to 1.01, with an average value of 0.34 (Figure 4A). Most organisms were close to the source, indicating that they occupied the same food web; however, the relative carbon sources for *T. kammalensis* and *T. mystax* were 1.01 and 0.82, respectively, indicating that their dietary source was similar to that of pelagics and included but was not limited to the food web shared by other organisms. In addition, the lipid concentration of SCCPs in *Valenciennes* and *Scapharca subcrenata* exceeded that of other organisms, possibly due to their benthic adhesion habits and feeding mainly on algae, plankton, and organic detritus. Moreover, adult Valenciennes fed on animal carcasses and lobobranchial species that might have been derived from additional dietary sources rather than the food web in this study. The TLs varied from 2.0 to 3.9 and followed the order of demersal predatory fish (3.75) > pelagic fish (3.82) > demersal omnivorous fishes (3.53) > cephalopods (3.51) > crustaceans (3.37) > shellfish (2.35) (Table 1).

In recent years, SCCP trophic transfer in aquatic food webs has attracted the attention of researchers [29]. A significant linear relationship (r^2^ = 0.76, *p* < 0.01) between TL values and the logarithmic ∑SCCPs (lipid-normalized) in the bottom food web was observed (Figure 4B). The TMF value was 2.19 to 3.01 (Table S5), indicating that a trophic transfer of SCCPs occurred from low to high TLs in the marine food webs in LZB. The results were similar to those obtained by Ma et al. [18] (2.38) and Huang et al. [24] (2.57) in Liaodong Bay and slightly lower than in the East China Sea (3.98) [43]. While, it was trophic dilution in a freshwater pond (TMFs = 0.17) and a region (TMFs = 0.238) in the Chinese Bohai Sea [19,25]. These differences may be caused by differences in the food web length [49], species vigor [32], metabolic elimination [50], and biotransformation [51] of SCCPs. For example, Zheng et al. [50] found that most Novel Brominated Flame Retardants (NBFRs,) which were similar to the SCCPs that were POPs, exhibited antimetabolic effects in fish with high TLs and significant trophic amplification, while only a few were metabolized rapidly in low trophic organisms and showed trophic dilution or insignificant trophic transfer behavior. In addition, the presence of compound biomagnification was not always observed because of the stochastic nature of predation in natural environments [46].

In terms of compounds, hydrophobic compounds with log K_ow_ values ranging from 5 to 8 demonstrate significant potential trophic amplification in aquatic food webs [52]. Significant positive linear relationships were observed between TMFs and log K_ow_ (r^2^ = 0.46, *p* < 0.01, Figure 4C), indicating that log K_ow_ may be a key controlling factor for the trophic transfer of SCCPs. In a freshwater food web [20], it is a parabolic curve (*p* < 0.01). A similar trend for NBFRs was observed in the South China Sea and was affected by biotransformation, which showed a significant negative relationship with TMFs [51]. Furthermore, the positive relationships were no longer observed when the hydrophobicity and steric hindrance increased because of the larger size of halogenated compounds [53]. In addition, a linear relationship between TMFs and carbon chain length was found (r^2^ = 0.64, *p* < 0.01, Figure 4D), which was similar to BAFs in a previous study [18,43,54]. This indicated that the carbon chain length may be a key factor for the trophic transfer of SCCPs. Previous researchers found differences in the metabolic rate of CPs in chicken liver microsomes based on carbon chain length. Moreover, this relationship was significantly greater than that based on chlorine content, which exhibited a sharp decline with increasing carbon chain length [55,56] and predator–prey relationships; therefore, the trophic transfer of SCCPs was regulated by log K_ow_ and carbon chain length in the marine food web in LZB. In addition, metabolic behavior may affect SCCP behavior.

### 3.5. Dietary Risk Assessment

EDI is the main path for human exposure to SCCPs [20], and marine food, as a main dietary food type among coastal urban populations, provides most SCCPs to the human body. Based on the average ∑SCCPs in marine organisms in LZB, the total daily intake of SCCPs for different age groups in Shandong Province was estimated (Table S6). The average total daily intake was 0.66 μg kg^−1^ d^−1^, and it was far lower than for the TDI (HQ < 1). These results indicated that in the current ΣSCCPs in the marine environment of LZB, there was no dietary risk; however, considering the persistence and bioaccumulation of SCCPs, the dietary risk of SCCPs is predicted to increase in the future.

## 4. Conclusions

SCCPs were widely distributed in water, sediments, and organisms in Laizhou Bay. Different from seawater and sediments, C_13_ and Cl_5–7_ (93.13%) were the most abundant congenant in organisms. The levels of SCCPs in the benthic species were much higher than those in the pelagic species, with the highest concentration in the shellfish, indicating that SCCPs in the benthic species were mainly derived from sediments. In the process of bioaccumulation, the chlorine content, carbon chain length, and log K_ow_ of SCCPs are the main control factors. In this process, with the increase of logkow and carbon chain length, TMFs increase, which was not observed with chlorine content. In the future, further studies are needed to understand the biotransformation, intestinal absorption, and metabolic elimination properties of SCCPs in ecosystems.

## Figures and Tables

**Table 1:** Basic information on the biological samples and short-chain chlorinated paraffins (SCCPs) ingested by the organisms.

Species	Sample Number	SCCP Concentrations			TL	LogBAF (L/kg w.w.)	BSAF (l.w./TOC)	Feeding Habits ^a^	Living Habitat ^b^
		(ng/g l.w.)	(ng/g d.w.)	(ng/g w.w.)					
**Shellfish**									
*Scapharca subcrenata*	3	18,446 ± 1771	163 ± 63	56 ± 21	2.43 ± 0.92	2.19	0.40	FL/DE	BE
*Rapana venosa*	7	24,756 ± 14,080	258 ± 126	59 ± 30	2.15 ± 0.56	2.15	0.50	OM/DE	BE
Average	-	26,247 ± 10,939	239 ± 121	75 ± 44	-	2.17	0.45	-	-
**Crustacean**									
*Palaemon gravieri*	3	4626 ± 846	49 ± 9	10 ± 2	3.55 ± 0.47	1.18	0.09	PL/OM	DE
*Oratosquilla oratoria*	9	3944 ± 3047	266 ± 221	53 ± 41	3.25 ± 1.45	2.17	0.08	OM	DE
*Alpheus japonicus Miers*	3	3554 ± 819	108 ± 25	25 ± 6	3.22 ± 0.49	1.84	0.07	OM	DE
*Fenneropenaeus chinensis*	4	2262 ± 1132	39 ± 34	9 ± 8	2.14 ± 0.32	1.39	0.03	OM	DE
*Scomber japonicus*	10	5455 ± 4544	279 ± 427	118 ± 180	3.47 ± 0.96	2.51	0.11	OM	DE
*Portunus trituberculatus*	5	3746 ± 872	383 ± 237	112 ± 36	3.59 ± 0.45	2.49	0.07	OM	DE
Average	-	3017 ± 2921	223 ± 278	66 ± 102	-	1.93	0.08	-	-
**Cephalopoda**									
*Octopusocellatus*	5	3769 ± 2039	109 ± 71	24 ± 13	3.44 ± 0.08	1.82	-	PL/OM	PE
*Loligo chinensis*	15	13,018 ± 7944	452 ± 367	82 ± 50	3.75 ± 0.2	2.35	-	PL/OM	PE
*Octopus variabilis*	3	7999 ± 1111	274 ± 38	50 ± 7	3.12 ± 0.16	2.14	-	PL/OM	PE
Average	-	9814 ± 7630	334 ± 330	62 ± 48	-	2.10	-	-	-
**Pelagic fishes**									
*Konosirus punctatus*	8	493 ± 494	316 ± 316	133 ± 133	3.63 ± 0.22	2.56	-	PL	PE
*Thryssa kammalensis*	5	361 ± 177	137 ± 67	40 ± 20	3.57 ± 0.13	2.07	-	PL	PE
*Thryssa mystax*	3	772 ± 599	351 ± 272	93 ± 72	3.77 ± 0.08	2.41	-	PL	PE
Average	-	520 ± 445	274 ± 258	98 ± 104	-	2.35	-	-	-
**Demersal fishes**									
*Pennahia argentata*	6	5815 ± 1693	867 ± 252	201 ± 59	3.61 ± 0.6	2.74	0.12	PD	DE
*Acanthogobius ommaturus*	7	4870 ± 6088	173 ± 224	35 ± 43	3.64 ± 0.25	1.98	0.10	PD	DE
*Cynoglossus semilaevis*	3	8772 ± 4198	69 ± 55	18 ± 9	3.53 ± 0.21	1.71	0.18	OM	DE
*Cynoglossus joyneri*	12	3443 ± 1997	266 ± 176	62 ± 36	3.55 ± 0.67	2.23	0.07	OM	DE
*Callionymidae*	6	11,574 ± 8586	103 ± 76	35 ± 26	3.82 ± 0.09	1.98	0.23	OM	DE
*Lateolabrax japonicus*	4	5281 ± 2801	124 ± 105	31 ± 16	3.97 ± 0.74	1.93	0.11	PD	DE
*Johnius grypotus*	4	1946 ± 331	244 ± 71	67 ± 11	3.61 ± 0.13	2.27	0.04	OM	DE
*Amblychaeturichthys hexanema*	3	3567 ± 814	160 ± 36	33 ± 8	3.39 ± 0.67	1.96	0.07	OM	DE
*Chaeturichthys stigmatias*	12	11,624 ± 8963	157 ± 100	37 ± 23	3.58 ± 0.68	2.01	0.23	OM	DE
*Platichthys bicoloratus*	4	6776 ± 6039	245 ± 219	52 ± 46	3.56 ± 0.58	2.16	0.14	OM	DE
*Takifugu niphobles*	4	3835 ± 2325	38 ± 24	9 ± 5	3.76 ± 0.26	1.39	0.08	OM	DE
*Platycephalus indicus*	5	8098 ± 6144	607 ± 460	118 ± 90	3.68 ± 0.58	2.51	0.16	PD	DE
*Saurida elongata*	3	4676 ± 1037	234 ± 52	54 ± 12	3.74 ± 1.12	2.17	0.09	OM	DE
*Tridentiger barbatus*	3	8663 ± 2033	202 ± 47	37 ± 9	3.48 ± 1.18	2.01	0.17	PD	DE
Average	-	6291 ± 5711	244 ± 252	56 ± 55	-	2.08	0.13	-	-

^a^ Feeding habits: Planktonic, PL; Detritivores, DE; Filtering, FL; Omnivorous, OM; Predatory, PD. ^b^ Living habitats: Benthonic, BE; Demersal, DE; Pelagic; PE.

## Data Availability

The data that support the findings of this study are available from the corresponding author upon reasonable request.

## Supplementary Materials

The following supporting information can be downloaded at: https://www.mdpi.com/article/10.3390/toxics12120877/s1, Text S1: Species name and comments name; Text S2: Chemical and materials; Text S3: Stable isotope analysis; Table S1: Acquisition Parameters for 24 SCCPs; Table S2: Biological parameters of components in the species from LZB; Table S3: Stable isotopes of aquatic species analyzed in this study; Table S4: The relationship between BSAFs and logKow of SCCPs; Table S5: The TMFs of SCCPs congener groups; Table S6 Dietary intake of SCCPs and their risk assessment; Figure S1: Relationship between SCCPs and the lipid concent of aquatic organism; Figure S2: The relationship between logBAFs and the carbon chain length in organisms; Figure S3: The relationship between logBAFs and the chlorine content in organisms; Figure S4: The relationship between logBAFs and logKow (A), and the logBAFs of Thryssa kammalensisi and Thryssa mystax with log Know; Figure S5: The relationship between BSAFs and the carbon chain length in organisms; Figure S6: The relationship between BSAFs and the chlorine content in organisms; Figure S7: Adjusted stable carbon (δ13C) and nitrogen (δ15N) isotope map for biota collected from Laizhou Bay; Figure S8: The relationship between TMFs and the chlorine content of SCCPs. References [57,58] are cited in the Supplementary Materials.
