# Supplementary material for: Occurrence, Bioaccumulation, and Trophic Transfer of Short-Chain Chlorinated Paraffins (SCCPs) in a Marine Food Web from Laizhou Bay, Bohai Sea (Eastern China)
Source: Toxics. 2024 Nov 30;12(12):877. doi: 10.3390/toxics12120877 (PMC11679791; doi:10.3390/toxics12120877)
Supplement: Supplementary file 1 [file toxics-12-00877-s001.zip › toxics-3271463-supplementary.pdf]

Occurrence, Bioaccumulation, and Trophic Transfer of Short-Chain Chlorinated Paraffins (SCCPs) in a Marine Food Web from Laizhou Bay, Bohai Sea (Eastern China)

Min Song [1], Dianfeng Han [2], Shunxin Hu [2], Qingkui Cui [2], Huanjun Li [2,*], Fan Li [2], Jianbai Zhang [3], Yongchun Liu [2], Mei Zhao [1], Cunxin Zhang [1] and Yingjiang Xu [2,*]

[1] School of Food, Shanghai Ocean University, Shanghai 200120, China
[2] Shandng Provincial Key Laboratory of Restoration for Marine Ecology, Yantai Key Laboratory of Quality and Safety Control and Deep Processing of Marine Food, Shandong Marine Resource and Environment Research Institute, Yantai 264006, China
[3] Yantai Marine Economic Research Institute, Yantai 264003, China
* Correspondence: lihuanjun_hyzhy@163.com (H.L.); sdsczj@shandong.cn (Y.X.)

List of the Supplementary Data

Text S1 Species name and comments name.(P.1)
Text S2 Chemical and materials. (P. 2)
Text S3 Stable isotope analysis. (P. 3-4)
Table S1 Acquisition Parameters for 24 SCCPs. (P. 5)
Table S2 Biological parameters of components in the species from LZB. (P. 6)
Table S3 Stable isotopes of aquatic species analyzed in this study. (P. 7-8)
Table S4 The relationship between BSAFs and logK_{ow} of SCCPs (P. 9)
Table S5 The TMFs of SCCPs congener groups. (P.10)
Table S6 Dietary intake of SCCPs and their risk assessment. (P. 10)
Figure S1 Relationship between SCCPs and the lipid concent of aquatic organism. (P. 11)
Figure S2 The relationship between logBAFs and the carbon chain length in organisms. (P. 12)
Figure S3 The relationship between logBAFs and the chlorine content in organisms. (P.12)
Figure S4 The relationship between logBAFs and logK_{ow} (A), and the logBAFs of Thryssa kammalensisi and Thryssa mystax with log K_{ow}. (P.13)
Figure S5 The relationship between BSAFs and the carbon chain length in organisms. (P.14)
Figure S6 The relationship between BSAFs and the chlorine content in organisms. (P.14)
Figure S7 Adjusted stable carbon ($\delta^{13}C$) and nitrogen ($\delta^{15}N$) isotope map for biota collected from Laizhou Bay. (P. 15)

Figure S1 The relationship between TMFs and the chlorine content of SCCPs.(P.15)

Text S1 Species name and comments name

The 28 species included shellfish, crustaceans, fish and cephalopods. The shellfish included rapana venosa (*Valenciennes*), blood clam (*Scapharca subcrenata*). The crustacean included four species of shrimp, the name as follows: palaemonoid shrimps (*Palaemon gravieri*), edible mantis shrimp (*Oratosquilla oratoria*), Japanes drum shrimp (*Alpheus japonicus Miers*), chinese shrimp (*Fenneropenaeus chinensis*), and two species of crabs included swimming crab (Portunus trituberculatus) and Charybdis japonica (*Scomber japonicus*). The cephalopods included shortarmoctopus (*Octopusocellatus*), squid (*Loligo chinensis*) and whiparm octopus (*Octopus variabilis*). The fish species included dotted gizzard Shad (*Konosirus punctatus*), kammal thryssa (*Thryssa kammalensis*), *Thryssa mystax*, Silver croaker (*Pennahia argentata*), marked lancettail, finespot goby (*Chaeturichthys stigmatias*), Tongue Sole (Cynoglossus semilaevis), Joyner's tongue-sole (*Cynoglossus joyneri*), *Callionymidae*, Japaneseseaperch (*Lateolabrax japonicus*), Johnius grypotus (*Johnius grypotus*), Pinkgray goby (*Amblychaeturichthys hexanema*), Acanthogobius ommaturus, Stone flounder (*Platichthys bicoloratus*), kusafugu (*Takifugu niphobles*), bartail flathead (*Platycephalus indicus*), lizardfish (*Saurida elongata*) and Tridentiger barbatus.

Text S2 Chemical and materials

Chromatographically pure acetonitrile (ACN), dichloromethane (DCM), methanol (MeOH), and n-hexane were purchased from Merck KGaA (Darmstadt, Germany). HLB solid-phase extraction columns (polystyrene-divinylbenzene, 60 mg, 3 mL) were purchased from the Waters Corporation (Milford, Massachusetts MA, USA). Glass cellulose filter membranes (0.45 μm) were purchased from Shanghai Xingya Products (Shanghai, China). Ammonia, sodium chloride, anhydrous sodium sulfate, and concentrated sulfuric acid were purchased from the Sinopharm Chemical Reagent Co . Sodium chloride and anhydrous sodium sulfate were baked at 600 °C for 6 h before use. Silica gel (63–100 μm) and Florisil (60–100 mesh) were purchased from Sigma-Aldrich (St. Louis, MO, USA). The silica gel was activated at 550 °C for 12 h before use, and Florisil was baked at 140 °C for 12 h before use. Activated silica gel (100 g) was thoroughly mixed with 23.3 ml of concentrated sulfuric acid to obtain acidified silica gel.

Text S3 Stable isotope analysis

Stable carbon isotope ($\delta^{13}C$) and nitrogen isotope ($\delta^{15}N$) ratios were used to determine the TL of an organism. The freeze–dried samples were ground and weighed (1-2mg) to analyze the $\delta^{13}C$ and $\delta^{15}N$ compositions by using an isotope ratio mass spectrometer (Thermo Delta V Advantage and Thermo Flash EA1112 HT) [35].

$$\delta^{15}N= [(^{15}N / ^{14}N_{sample}) / (^{15}N / ^{14}N_{standard}) - 1]\times1000 \quad (A.1)$$

$$\delta^{13}C= [(^{13}C / ^{12}C_{sample}) / (^{13}C / ^{12}C_{standard}) - 1]\times1000 \quad (A.2)$$

To avoid the influence of lipid content on $\delta^{13}C$ values, the following formula was used to normalize $\delta^{13}C$ [57]:

$$\delta^{13}C_{normalized} = \delta^{13}C_{untreated} - 3.32 + 0.99 \times C/N \quad (A.3)$$

The relative carbon sources are closely related to their feeding environment and

species, which can be used to assess whether samples belong to the same food web; when close to 0, it indicates that they are a bottom feeder, and when close to 1, it indicates they are an upper–middle feeder [58]. The calculation formula is as follows:

$$\text{Carbon source} = 1 - \frac{\delta^{13}C_{zooplankton} - \delta^{13}C_{consumer} + \Delta\delta^{13}C(TL_{consumer} - TL_{bentic})}{\delta^{13}C_{zooplankton} - \delta^{13}C_{bentic}} \quad (A.4)$$

It is assumed that $\delta^{13}C_{zooplankton}$ are aquatic animals with a plankton diet, representing a pelagic source. $\delta^{13}C_{bentic}$ represents benthic sources, and $\Delta^{13}C$ is a constant nutrient enrichment factor for consumers with a value of 1.3‰.[2]

Reference

35. Li, H.; Zhang, Z.; Sun, Y.; Wang, W.; Xie, J.; Xie, C.; Hu, Y.; Gao, Y.; Xu, X.; Luo, X.; Mai, B. Tetrabromobisphenol A and hexabromocyclododecanes in sediments and biota from two typical mangrove wetlands of South China: Distribution, bioaccumulation and biomagnification. Sci. Total Environ. 2021, 750, 141695, DOI: 10.1016/j.scitotenv.2020.141695.

57. Liu, S. S.; Zhao, H. X.; Lehmler, H. J.; Cai, X. Y.; Chen, J. W. Antibiotic Pollution in Marine Food Webs in Laizhou Bay, North China: Trophodynamics and Human Exposure Implication. Environ. Sci. Technol. 2017, 51 (4), 2392-2400, DOI: 10.1021/acs.est.6b04556.

58. McKinney, M. A.; McMeans, B. C.; Tomy, G. T.; Rosenberg, B.; Ferguson, S. H.; Morris, A.; Muir, D. C. G.; Fisk, A. T. Trophic Transfer of Contaminants in a Changing Arctic Marine Food Web: Cumberland Sound, Nunavut, Canada. Environ. Sci. Technol. 2012, 46 (18), 9914-9922, DOI: 10.1021/es302761p.

Table S1 Biological parameters of components in the species from LZB.

Sample	Body length range(mm)	Weight range(g)	Water content (%)	Fat content (%)
Shellfish				
Scapharca subcrenata	-	0.1-20.9	66.00	0.21
Rapana venosa	-	27.3-176.5	77.01	0.24
Crustacean				
Palaemon gravieri	4.0-5.3	0.7-2.1	79.03	0.22
Oratosquilla oratoria	6.4-16.3	3-47.2	80.81	1.36
Alpheus japonicus Miers	2.5-6.8	0.1-4.6	77.00	0.67
Fenneropenaeus chinensis	8.7-14.3	25.5-28.0	77.00	0.70
Charybdis japonica	3.6-7.8	9.0-81.0	75.76	3.96
Portunus trituberculatus	3.8-16.5	2.8-222.2	75.62	4.09
Demersal fishes				
Pennahia argentata	12.3-15.8	36.1-72.3	76.81	8.06
Acanthogobius ommaturus	13.3-28	13.9-107.5	80.48	0.71
Cynoglossus semilaevis	4.0-27.0	21.1-110.4	79.39	0.21
Cynoglossus joyneri	8.5-17.3	3.0-28.0	77.79	2.43
Callionymidae	7.1-12	2.1-8.6	82.68	0.30
Lateolabrax japonicus	12.8-24.5	37.1-182.4	78.60	0.59
Johnius grypotus	7.0-8.8	4.7-6.6	76.81	3.46
Amblychaeturichthys hexanema	5.8-15.3	1.8-27.4	79.25	0.93
Chaeturichthys stigmatias	5.6-22.8	1.0-68.4	80.95	0.25
Platichthys bicoloratus	12.5-15.8	20.0-49.4	78.86	0.77
Takifugu niphobles	8.4-13.2	24.5-52.2	78.56	0.23
Platycephalus indicus	21-25.3	40.5-81.3	80.52	1.46
Saurida elongata	10.7-11.2	18.6-27.9	81.57	0.43
Tridentiger barbatus	13.6-26.3	19.8-127.0	77.03	1.11
Pelagic fishes				
Konosirus punctatus	12-18.5	23.9-114.6	57.91	26.97
Thryssa kammalensis	5.0-6.9	0.9-4.6	70.68	11.10
Thryssa mystax	10-13.1	7.4-18.1	73.51	12.03
Cephalopoda				
Octopusocellatus	4.0-6.8	17.1-59	81.60	0.63
Loligo chinensis	1.6-17.6	0.3-5.3	85.03	0.63
Octopus variabilis	6.7-8.2	60.5-79.9	81.60	0.63

Table S2 Acquisition Parameters for 24 SCCPs.

Congener group	Formula	Ion	Quantifier (m/z)	Qualifier 1 (m/z)	Qualifier 2 (m/z)
C_{10}	$C_{10}H_{17}Cl_5$	$[M+Cl]^-$	348.94264	350.93969	346.94559
	$C_{10}H_{16}Cl_6$	$[M+Cl]^-$	382.90367	384.90072	386.89777
	$C_{10}H_{15}Cl_7$	$[M+Cl]^-$	418.86175	416.8647	420.8588
	$C_{10}H_{14}C_{l8}$	$[M+Cl]^-$	452.82278	450.82573	454.81983
	$C_{10}H_{13}Cl_9$	$[M+Cl]^-$	486.7838	488.78085	484.78675
	$C_{10}H_{12}Cl_{10}$	$[M+Cl]^-$	520.74483	522.74188	518.74778
C_{11}	$C_{11}H_{19}Cl_5$	$[M+Cl]^-$	362.95829	364.95534	360.96124
	$C_{11}H_{18}Cl_6$	$[M+Cl]^-$	396.91932	398.91637	400.91342
	$C_{11}H_{17}Cl_7$	$[M+Cl]^-$	432.8774	430.88035	434.87445
	$C_{11}H_{16}Cl_8$	$[M+Cl]^-$	466.83843	464.84138	468.83548
	$C_{11}H_{15}Cl_9$	$[M+Cl]^-$	500.79945	502.7965	498.8024
	$C_{11}H_{14}Cl_{10}$	$[M+Cl]^-$	534.76048	536.75753	532.76343
C_{12}	$C_{12}H_{21}Cl_5$	$[M+Cl]^-$	376.97394	378.97099	374.97689
	$C_{12}H_{20}Cl_6$	$[M+Cl]^-$	410.93497	412.93202	414.93873
	$C_{12}H_{19}Cl_7$	$[M+Cl]^-$	446.89305	444.896	448.8901
	$C_{12}H_{18}Cl_8$	$[M+Cl]^-$	480.85408	478.85703	482.85113
	$C_{12}H_{17}Cl_9$	$[M+Cl]^-$	514.8151	516.82181	512.81805
	$C_{12}H_{16}Cl_{10}$	$[M+Cl]^-$	548.77613	550.77318	546.77908
C_{13}	$C_{13}H_{23}Cl_5$	$[M+Cl]^-$	390.98959	392.98664	388.99254
	$C_{13}H_{22}Cl_6$	$[M+Cl]^-$	424.95062	426.94767	428.94472
	$C_{13}H_{21}Cl_7$	$[M+Cl]^-$	460.9087	458.91165	462.90575
	$C_{13}H_{20}Cl_8$	$[M+Cl]^-$	494.86973	492.87268	496.86678
	$C_{13}H_{19}Cl_9$	$[M+Cl]^-$	528.83075	530.8278	526.8337
	$C_{13}H_{18}Cl_{10}$	$[M+Cl]^-$	562.79178	564.78883	560.79473

Table S3 The relationship between BSAFs and logK_{ow} of SCCPs

Compound	BSAF=a＋b×log K_{ow}	r^2	p-value
Scapharca subcrenata	y = −1.44＋8.68	0.40	<0.05
Rapana venosa	y = −1.01x + 6.05	0.57	<0.01
Palaemon gravieri	y = −0.16x + 0.93	0.45	<0.01
Oratosquilla oratoria	y = −0.22x + 1.34	0.40	<0.05
Alpheus japonicus Miers	y = −0.20x + 1.22	0.38	<0.05
Charybdis japonica	y = −0.29x + 1.74	0.42	<0.01
Portunus trituberculatus	y = −0.19x + 1.16	0.44	<0.01
Pennahia argentata	y = −0.34x + 2.06	0.44	<0.01
Acanthogobius ommaturus	y = −0.35x + 2.09	0.50	<0.01
Cynoglossus semilaevis	y = −0.54x + 3.27	0.42	<0.01
Cynoglossus joyneri	y = −0.25x + 1.48	0.51	<0.01
Callionymidae	y = −0.76x + 4.57	0.42	<0.01
Lateolabrax japonicus	y = −0.42x + 2.52	0.59	<0.01
Johnius grypotus	y = −0.11x + 0.69	0.47	<0.01
Amblychaeturichthys hexanema	y = −0.18x + 1.08	0.35	<0.05
Chaeturichthys stigmatias	y = −0.74x + 4.44	0.46	<0.01
Platichthys bicoloratus	y = −0.38x + 2.27	0.42	<0.05
Takifugu niphobles	y = −0.48x + 2.89	0.47	<0.01
Platycephalus indicus	y = −0.31x + 1.87	0.45	<0.01
Saurida elongata	y = −0.51x + 3.05	0.48	<0.01

Table S4 Stable isotopes of aquatic species analyzed in this study.

Sample	δ¹⁵N ‰	Content of N(%)	δ¹³C ‰	Content of C (%)	C/N	Adjusted δ¹³C ‰	Relative carbon source
Shellfish							
Scapharca subcrenata	8.91±0.35	10.74	-18.8±0.23	49.06	4.4549	-18.52±0.28	0.19±0.06
Rapana venosa	7.85±0.21	8.86±1.60	-19.81±0.37	39.45±1.38	4.49±0.97	-18.66	-0.05±0.12
Crustacean							
Palaemon gravieri	13.17±0.18	13.31±0.82	-19.59±0.6	46.02±2.06	3.46±0.13		0.27±0.18
Oratosquilla oratoria	12.06±0.32	11.33±1.57	-18.98±0.4	43.57±1.62	3.91±0.55	-19.04±0.82	0.18±0.37
Alpheus japonicus Miers	11.93±0.18	9.73±1.47	-19.13±0.95	39.36±2.97	4.11±0.65	-18.67±0.33	0.02±0.15
Fenneropenaeus chinensis	6.74±0	11.85±0.07	-20.49±0	41.41±0.16	3.5±0.03	-20.17±0.26	0.68±0.12
Charybdis japonica	12.5±0.23	10.34±1.84	-20.24±1.17	41.58±1.43	4.26±0.79	-19.11±0.77	0.21±0.34
Portunus trituberculatus	13.34±0.17	11.63±1.20	-19.99±0.7	45.93±3.07	3.96±0.17	-19.54±0.2	0.41±0.09
Demersal fishes							
Pennahia argentata	13.38±0.05	14.38±0.95	-19.56±0.13	51.13±0.59	3.56±0.28	-19.36±0.15	0.32±0.07
Acanthogobius ommaturus	13.55±0.09	13.48±0.78	-19.5±1.41	43.87±1.14	3.27±0.2	-19.79±0.29	0.52±0.13
Cynoglossus semilaevis	11.72±0.08	15.155±0.56	-17.93±0.14	46.46±0.53	3.07±0.08	-18.21±0.3	-0.19±0.13
Cynoglossus joyneri	13.18±0.25	13.58±1.38	-19.6±1.55	45.11±2.43	3.34±0.19	-19.62±0.67	0.44±0.3
Callionymidae	14.22±0.03	14.26±1.07	-20.31±0.07	46.21±0.39	3.25±0.24	-20.05±0.21	0.63±0.1
Lateolabrax japonicus	13.41±0.23	14.31±0.33	-18.58±0.19	46.40±0.31	3.24±0.06	-19.12±0.17	0.22±0.08
Johnius grypotus	12.58±0.23	13.69±1.32	-19.63±0.28	45.71±2.23	3.35±0.22	-19.43±0.24	0.36±0.11
Amblychaeturichthys hexanema	13.3±0.26	13.27±1.43	-19.67±0.92	44.20±2.25	3.35±0.18	-19.53±0.47	0.4±0.21

Sample	δ^{15}N ‰	Content of N(%)	δ^{13}C ‰	Content of C (%)	C/N	Adjusted δ^{13}C ‰	Relative carbon source
Chaeturichthys stigmatias	13.91±0.42	13.51±2.11	-18.74±0.44	35.73±1.95	2.75±1.17	-19.65±0.52	0.45±0.23
Platichthys bicoloratus	13.22±0.22	13.64±1.00	-19.6±0.45	45.99±0.54	3.38±0.2	-19.59±0.8	0.43±0.35
Takifugu niphobles	13.97±0.1	14.12±1.48	-19.61±0.32	46.31±2.41	3.29±0.19	-19.42±0.11	0.35±0.05
Platycephalus indicus	13.7±0.22	14.11±1.83	-18.94±1.01	46.49±2.80	3.32±0.25	-18.86±0.28	0.1±0.12
Saurida elongata	12.9±0.45	12.87±1.81	-19.51±1.12	43.56±1.78	3.43±0.38	-19.55±0.24	0.41±0.11
Tridentiger barbatus	14.77±0.27	13.54±1.47	-20.16±0.76	46.2233±1.55	3.48±0.59	-19.47±0.67	0.37±0.3
pelagic fishes							
Konosirus punctatus	11.82±0.15	7.80±0.78	-19.48±0.13	55.79±1.14	7.2±0.8	-19.46±0.34	0.37±0.15
Thryssa kammalensis	13.26±0.48	12.83±1.03	-20.35±0.76	44.62±1.89	3.48±0.35	-20.88±0.65	1.01±0.29
Thryssa mystax	14±0.27	10.86±0.92	-21.22±4.93	56.72±1.24	5.26±0.84	-20.46±0.03	0.82±0.01
Cephalopoda							
Octopusocellatus	12.78±0.31	11.93±1.74	-18.94±0.72	42.47±1.22	3.56±0.17	-19.21±0.03	0.26±0.01
Loligo chinensis	13.93±0.77	12.06±1.23	-19.09±0.62	44.40±1.44	3.71±0.33	-19.69±0.22	0.47±0.1
Octopus variabilis	11.54±0.61	11.06±1.17	-19.16±0.47	39.93±1.52	3.62±0.16	-18.69±0.32	0.02±0.14

Table S1 The TMFs of SCCPs congener groups

SCCPs	log Kow	TMFs			
		Regression equation	*r*	*p*	Value
$C_{10}H_{17}Cl_5$	5.09	y = 0.340x + 1.206	0.631	<0.01	2.188
$C_{10}H_{16}Cl_6$	5.22	y = 0.355x + 1.478	0.629	<0.01	2.264
$C_{10}H_{15}Cl_7$	5.42	y = 0.346x + 1.002	0.617	<0.01	2.218
$C_{10}H_{14}Cl_8$	5.65	y = 0.478x - 0.454	0.743	<0.01	3.006
$C_{11}H_{19}Cl_5$	5.24	y = 0.377x + 1.303	0.653	<0.01	2.382
$C_{11}H_{18}Cl_6$	5.43	y = 0.396x + 1.330	0.67	<0.01	2.489
$C_{11}H_{17}Cl_7$	5.56	y = 0.364x + 1.127	0.681	<0.01	2.312
$C_{11}H_{16}Cl_8$	5.68	y = 0.356x + 0.382	0.425	>0.05	2.27
$C_{12}H_{21}Cl_5$	5.64	y = 0.442x + 0.962	0.628	<0.01	2.767
$C_{12}H_{20}Cl_6$	5.66	y = 0.382x + 1.246	0.687	<0.01	2.41
$C_{12}H_{19}Cl_7$	5.76	y = 0.377x + 0.979	0.709	<0.01	2.382
$C_{12}H_{18}Cl_8$	5.97	y = 0.413x + 0.263	0.72	<0.01	2.588
$C_{13}H_{23}Cl_5$	5.98	y = 0.461x + 1.066	0.743	<0.01	2.891
$C_{13}H_{22}Cl_6$	5.76	y = 0.416x + 1.266	0.735	<0.01	2.606
$C_{13}H_{21}Cl_7$	5.89	y = 0.405x + 1.211	0.725	<0.01	2.541
$C_{13}H_{20}Cl_8$	6.04	y = 0.420x + 0.810	0.738	<0.01	2.63

Table S2 Dietary intake of SCCPs and their risk assessment

	EDI(ng/(kg.d)		HQ(ng/(kg.d)	
age	man	woman	man	woman
2~7	66.64	76.15	<0.01	<0.01
8~12	69.29	71.20	<0.01	<0.01
13~19	55.80	50.34	<0.01	<0.01
20~50	67.59	66.99	<0.01	<0.01
51~65	71.54	67.91	<0.01	<0.01
>65	84.89	72.53	<0.01	<0.01

Figure S1

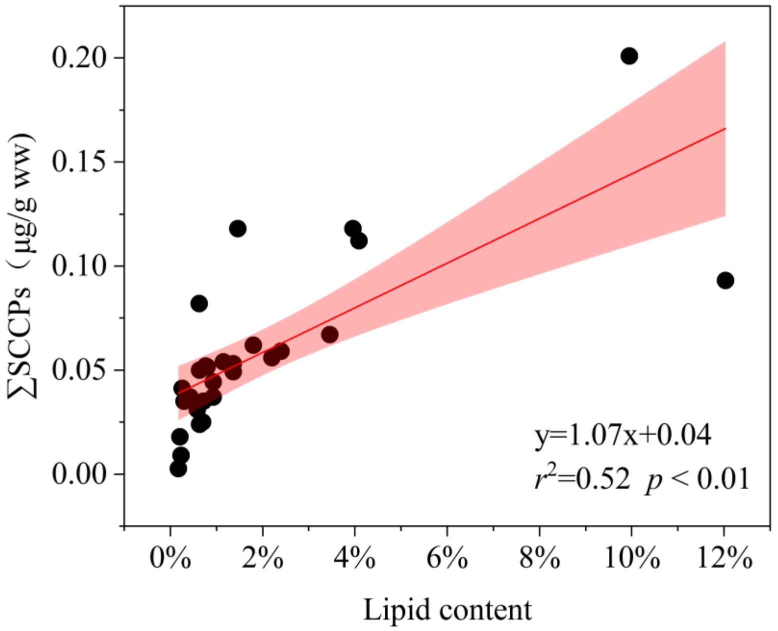

Figure S1 Relationship between SCCPs and the lipid content of aquatic organism

Figure S2

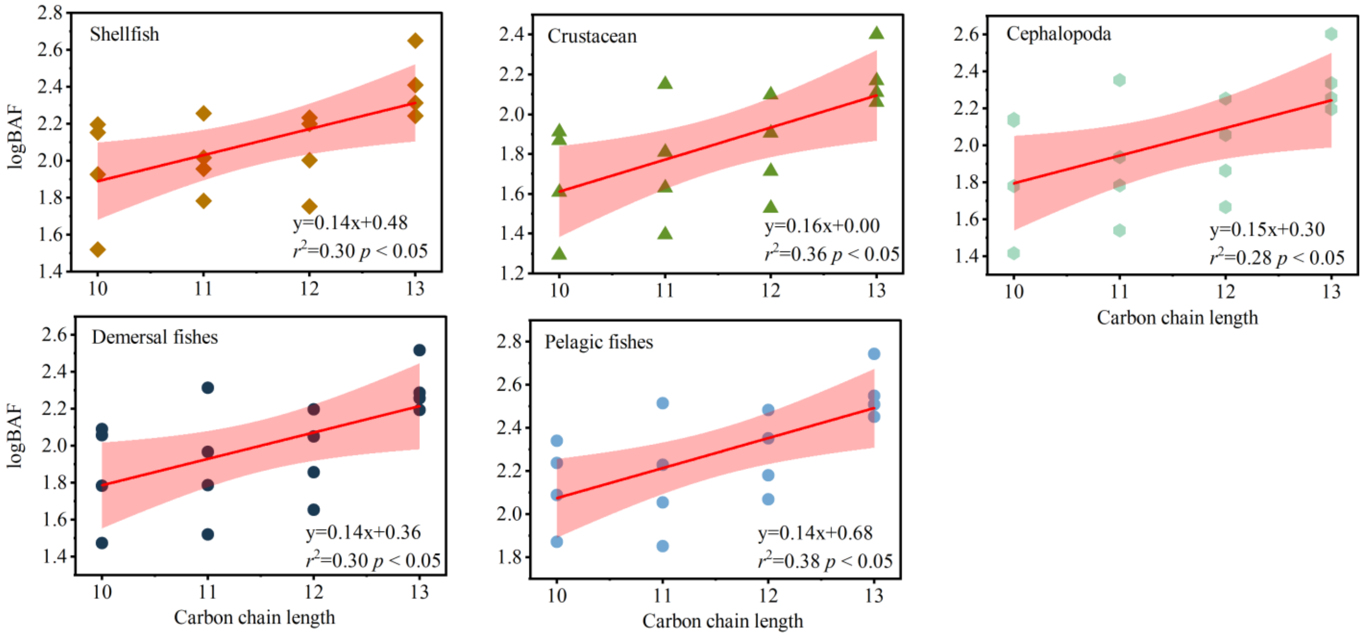

Figure S2 The relationship between logBAFs and the carbon chain length in organisms

Figure S3

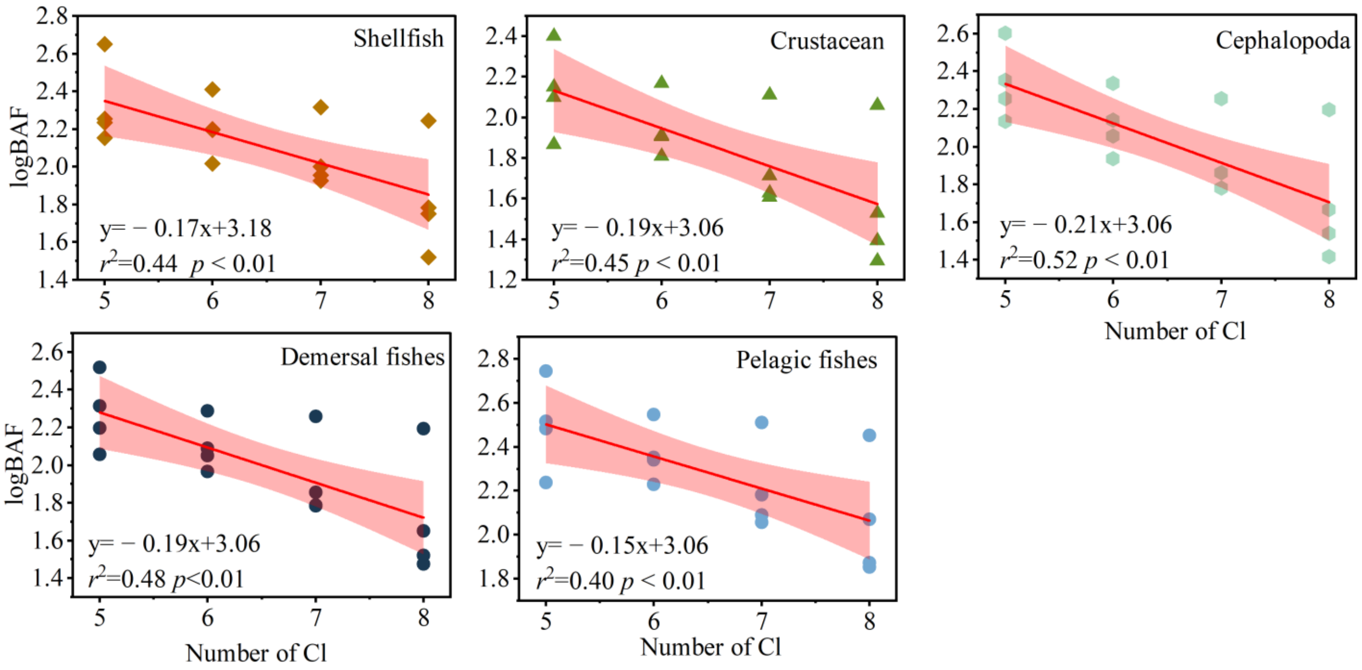

Figure S3 The relationship between logBAFs and the chlorine content in organisms

Figure S4

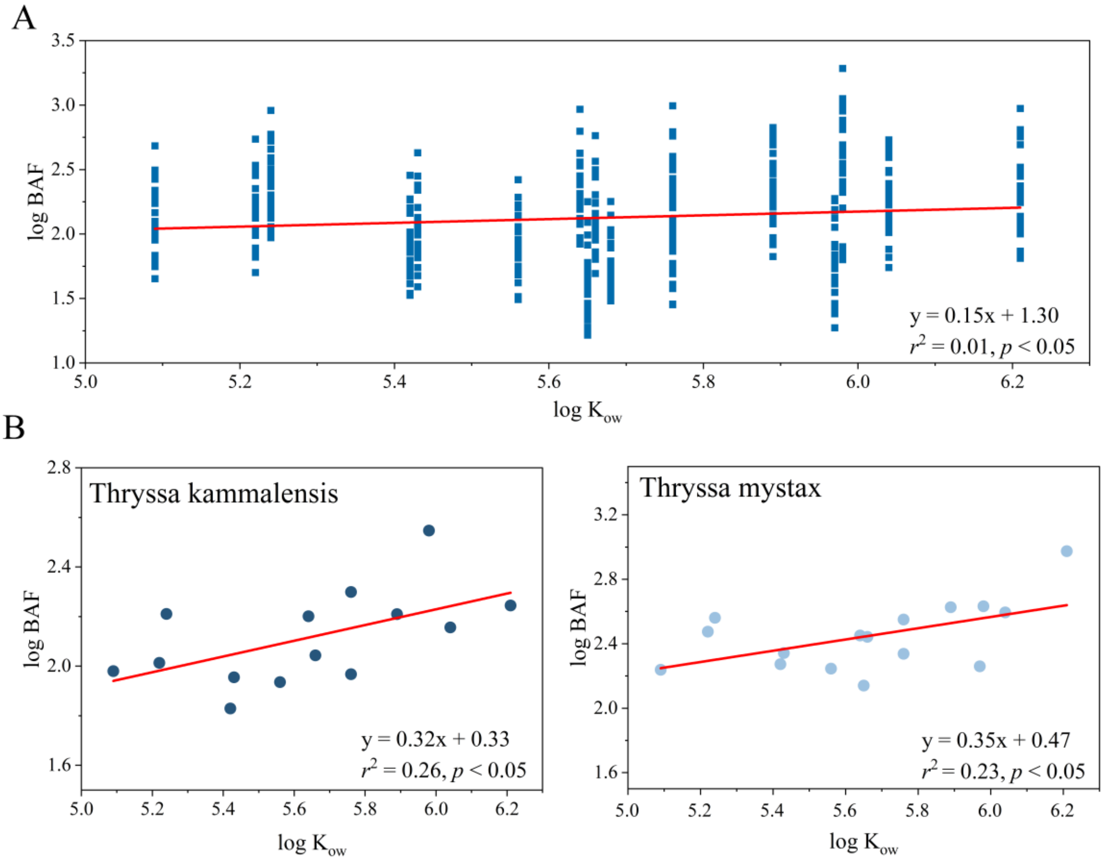

Figure S4 The relationship between logBAFs and logK_{ow} (A), and the logBAFs of Thryssa kammalensisi and Thryssa mystax with log K_{ow}

Figure S5

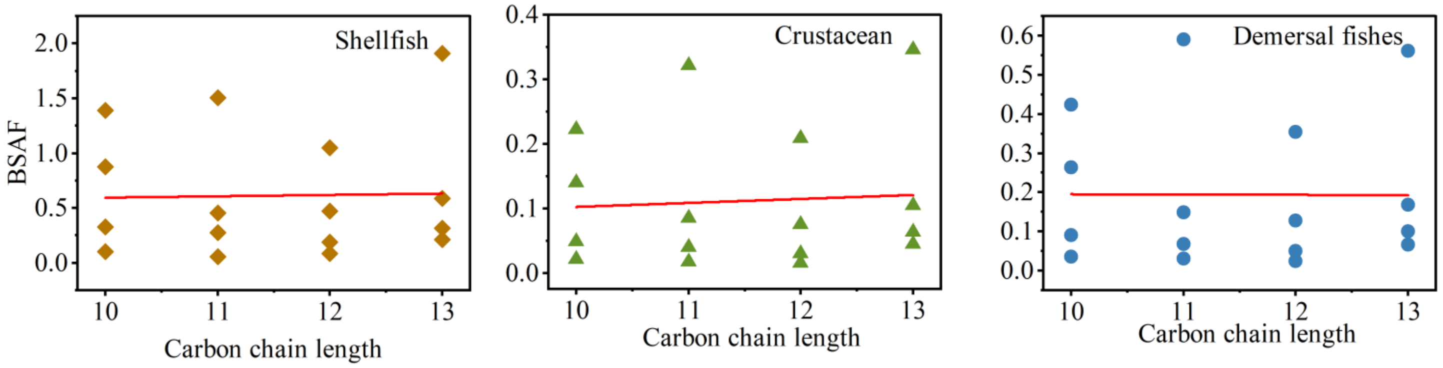

Figure S5 The relationship between BSAFs and the carbon chain length in organisms

Figure S6

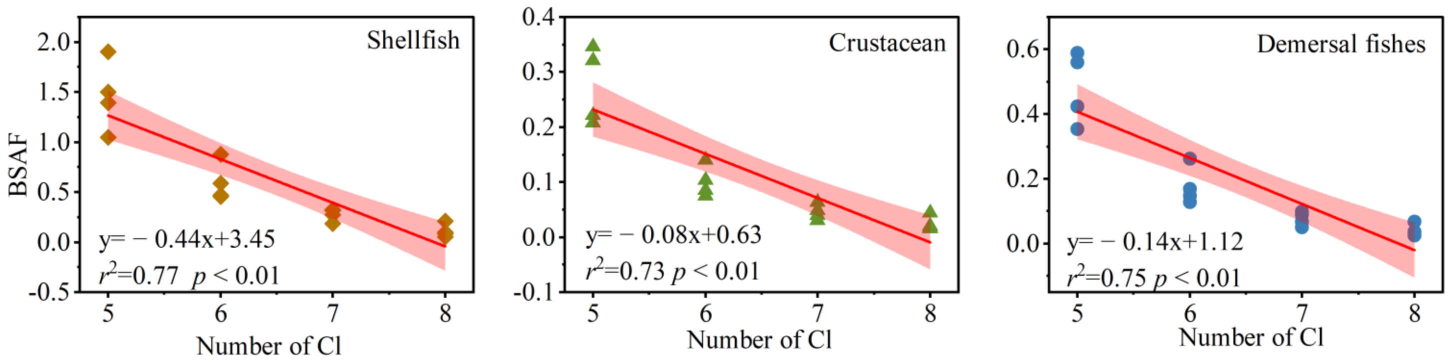

Figure S6 The relationship between BSAFs and the chlorine content in organisms

Figure S7

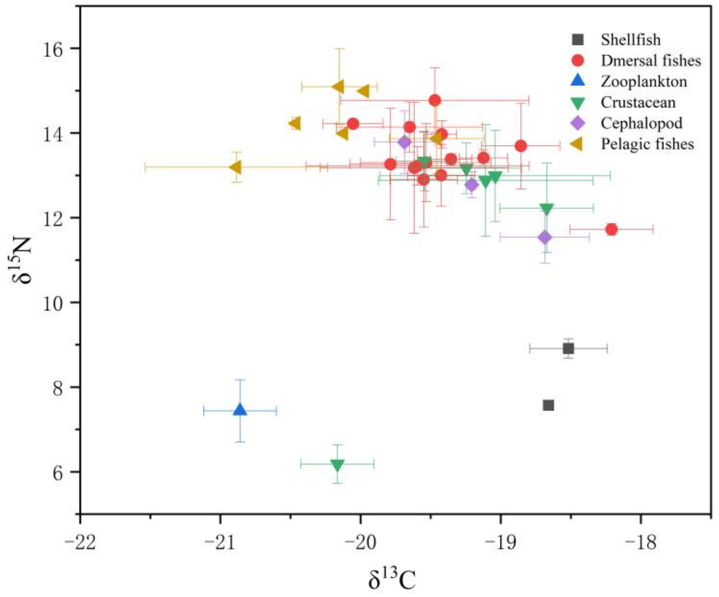

Figure S7 Adjusted stable carbon (δ^{13}C) and nitrogen (δ^{15}N) isotope map for biota collected from Laizhou Bay.

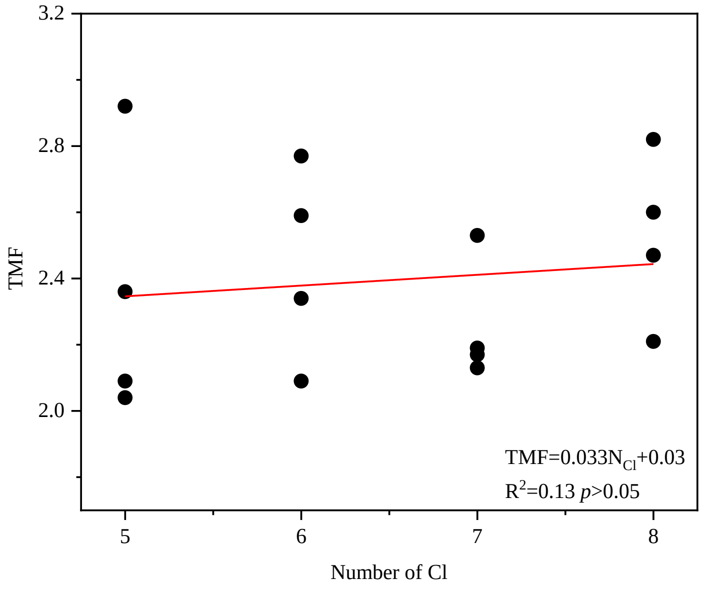

Figure S2 The relationship between TMFs and the chlorine content of SCCPs.
